# Comparison of the shear bond strength of 3D printed temporary bridges materials, on different types of resin cements and surface treatment

**DOI:** 10.4317/jced.55617

**Published:** 2019-04-01

**Authors:** Lorenz Holmer, Ahmed Othman, Anne-Katrin Lührs, Constantin von See

**Affiliations:** 1 Dr. med. dent student, Danube Private University, Krems an der Donau, Austria; 2 Dr., M.Sc., Digital Orthodontic Researcher, Digital technology in dentistry and CAD/CAM department, Danube Private University, Krems an der Donau, Austria; 3 DDS, PhD, Senior Lecturer, Department of Conservative Dentistry, Periodontology and Preventive Dentistry, Hannover Medical School, Hannover; 4 Prof. Dr., Director of the Center for Digital Technologies in Dentistry and CAD/CAM, Danube Private University, Krems an der Donau, Austria

## Abstract

**Background:**

Thus, purpose of this study was to compare the shear bond strength of the resin cement and the resin modified glass ionomer cement on 3D printed temporary material for crowns and bridges in combination with different surface treatment modalities.

**Material and Methods:**

Test specimens VarseoSmile Temp material (Bego, Bremen, Germany) (n=64) in the form of rectangular blocks (n=32) and cylindrical test specimens (n=32) were printed using the Varseo S 3D printer (Bego, Bremen, Germany). The specimens were divided into 4 groups, with 8 specimens of each kind. Two groups (n=16 pairs) were blasted with Perlablast® Micro [PM] 50µm (Bego, Bremen, Germany) and two groups (n=16 pairs) were blasted with alumina [AL] 50µm. The cylindric specimen were cemented on the rectangular block with a load of 20N using a Zwick/Roell machine (Ulm, Germany), to ensure a comparable cementing process. One group (n=8) of each pre-treatment was cemented with Fuji Cem 2 [Fuji+AL & Fuji+PM] and one of each with Variolink® Esthetic [Vario+AL & Vario+PM]. The Fuji Cem 2 was chemically cured while dual curing Variolink® Esthetic was additionally light cured using LED (Bluephase II, Ivoclar Vivadent, Ellwagen, Germany; light intensity, >1,000 mW/cm2, high power modus). The shear strength was performed with Zwick/Roell universal test machine (speed, 0.8 mm/min), fracture and statistical analysis was performed (T-test, *p*<0.05).

**Results:**

T-test showed a significant difference Fuji Cem 2 (Fuji+AL & Fuji&PM) and Variolink® Esthetic (Vario+AL &Vario+PM) (*p*=0.000). Fuji+AL & Fuji+PM showed a significant difference for surface pre-treatment (*p*=0.002). Vario+AL & Vario+PM no significance (*p*=0.872) for pre-treatment method was detectable.

**Conclusions:**

Variolink® Esthetic showed a higher bond strength compared to Fuji Cem 2 and an increasing bond strength for Fuji Cem 2 with alumina pre-treatment. There was no significant difference for Vario+AL and Vario+PM.

** Key words:**Shear bond strength, adhesion, adhesive resin cement, resin modified glass ionomer cement, 3D printable materials, mechanical testing, provisional restoration.

## Introduction

The advances in dentistry and the positive development of oral hygiene of the most patients have led to a longer maintenance of one’s own teeth and thus to an increased demand for fixed prosthetic restorations such as crowns and bridges. The resulting gain in the demand for temporary materials is awakening new technologies and materials, which have to meet all requirements. Among others, temporary materials must protect the mechanical tooth stability, restore phonetics, mastication, aesthetics and withstand mastication forces (1). The prerequisite for fulfilling all requirements is a secure bond between material and the tooth. The necessary requirements are influenced by the individual design of each temporary restoration, but the resistance to chewing forces and the ability to attach a material to teeth are material-specific properties, which must be tested *in vitro* prior to clinical application in the laboratory.

Manufacturing temporary: chairside by hand, milling, printing

As the materials have specific mechanical and chemical behaviour the bonding has to be estimated specifically for each material.

In general, the luting materials can be divided into two groups, the passive materials (zinc phosphate cement, zinc polycarboxylate, glass ionomer and resin- modified glass ionomer luting materials), which are bonding by mechanical friction and mechanical wedging. Chemical or adhesive materials can interact with tooth surfaces and prosthodontic materials, that allows a functional connection of restoration and tooth which leads to a reinforcement of the tooth and restoration (2,3). Adhesive luting materials have a significantly higher bond strength after a 14-day water bath and subsequent thermocycling to high-gold-content alloy material and aluminium oxide ceramics (4). Beside chemical bonding the bond strength can be affected by several surface pre-treatments 5. In fact Blixt *et al.* found that glass ionomer cements have a higher adhesion to aluminium oxide ceramic with previous surface treatment with 110μm alumina particles at 2.8 bar for 13 seconds (5).

As 3D printing resin for temporary crowns and bridges have been recently developed, there are only limited data and studies on bond strength of various luting materials or different surface pre-treatments available. The aim of this study was to analyse the shear bond of different materials in connection with different surface pre-treatment on printable crown and bridges materials.

## Material and Methods

Thirty-two specimens were 3D printed using Varseo S printer (Bego, Bremen, Germany) with DLP technology (digital light processing) with VarseoSmile Temp A2 for this study. The specimens were designed using a computer-aided program (Autodesk Netfabb, San Rafael, CA, USA). The post processing of the specimens was performed according to the manufacturer’s instructions. Unheated ultrasonic reusable ethanol jar with concentration 96% was used to clean the specimens for 3 minutes followed by 2 more minutes of a new ethanol bath with 96% concentration. The specimens were withdrawn from the ethanol bath and dried with compressed air. After eliminating all specimens’ printable supports the specimens were randomly divided into four experimental groups (n=8 pairs) ( Table 1). Groups were characterized by pre-treatment method and/or different resin used for bonding. The prospective bonding surfaces were pre-treated with alumina 50µm [AL] (n=16) or with Perlablast® Micro 50µm [PM] (n=16) from 1cm distance, an angle of 45° and 1,5 bar pressure (6). All test bodies were cleaned with compressed air. Surface polymerization using Nitrogen gas (1.0-1.2 bar) pressed into Otoflash (Bego, Bremen, Germany) with 10 light frequency/ second. Two periods with 1500 flashes were made and the samples were upturned after the first 1500 flashes.

**Table 1:**
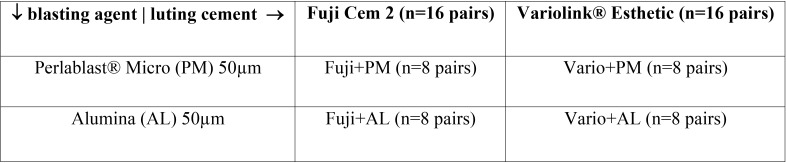
Overview of the group classification.

For every cylindrically specimens the diameter was measured on three different points with an outside micrometer (Mitutoyo, Kawasaki, Japan), to enable a correct calculation of the prospective bonding surface. The three diameters were averaged as surface area.

In this study, two different cements were used: Fuji Cem 2 [Fuji] (GC, Tokyo, Japan) for the first two groups and Variolink® Esthetic [Vario] (Ivoclar Vivadent, Schaan, Liechtenstein) for the other two groups ( Table 1). The cementation was performed using 20 N loading forces on the specimens (7,8).

Fuji consists of two main components which are an alumino-fluro-silicate glass, which is the base, and a polyacrilic acid acting as the catalyst (9). It was used with an automix despender and the first mixed material was discarded. A mixed portion was applied on the surfaces of both specimens. The application of the cement was done with the system’s own mixing tip. The specimens were then immediately loaded in the universal testing machine (Z010 Zwick/Roell, Ulm, Germany), and the excess material was removed with microbrushes (Micro Applicator brush, Ultradent Products, South Jordan, United States) directly after loading.

While, the Vario has main components which are Initiators (Ivocerin®) and additives. Monobond Plus (Ivoclar Vivadent, Ellwagen, Germany) was applied on the bonding surfaces of all specimens from the two Vario groups according to manufactures instructions. After drying with compressed air, all specimens (n=16) of the two groups were moistened with Variolink® Esthetic DC neutral. The Variolink® system was also used with original automix tip. The specimens were also loaded with 20N during the bonding process (7,8), after removing excess material with microbrush and applying Liquid Strip (Ivoclar Vivadent, Schaan, Liechtenstein) to protect the resin from oxygen inhibition, Vario was light cured for 15 seconds from four sides with Bluephase 20i (Ivoclar Vivadent, Ellwagen, Germany) in high power mode with 1200mW/cm2. For all Vario specimens [Vario+AL & Vario+PM], the tip of the polymerization unit was placed in direct contact to the specimens.

All specimens were loaded for 6 minutes while bonding. Afterwards, the shear bond strength test was performed with a universal testing machine Z010 (Zwick/Roell, Ulm, Germany) at a crosshead speed of 0,8 mm/min (8). According to the ISO 11405/2003 as recommended to 0.75 +/- 0.3 mm/min (10). The shear stamp made of hardened steel hit the test piece immediately next to the cement joint in a parallel direction of force to the adhesive surface of the test body (8). All results were recorded in N (Newton) and later converted into MPa (MegaPascal). The maximum values for each specimen were used as bond strength.

**Figure 1:**
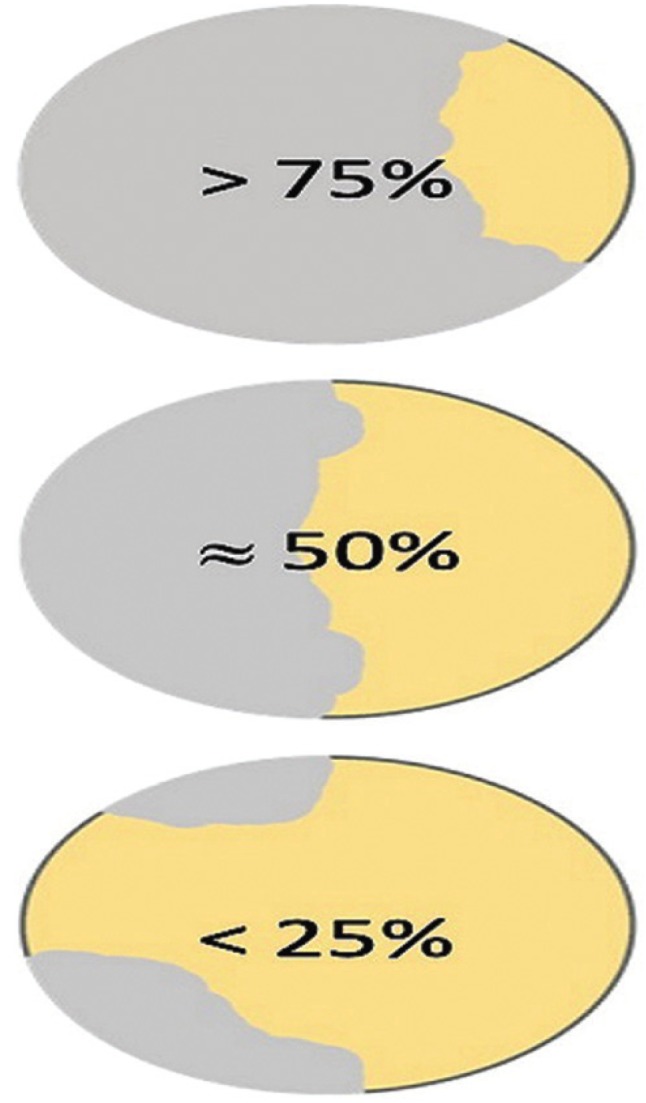
Division fracture surface.

-Descriptive analysis:

Fracture surfaces were divided in three groups: adhesive failure (> 75% of surface are showed a loss of bond), mixed failure (about 50% of surface showed a failure of bond and a failure of substrate), substrate failure (< 25% of surface area showed a loss of bond) (Fig. 1). An optical classification was carried out with the help of a magnifying lamp (Maul, Bad König, Germany) with 1.75-fold magnification (11).

Statistical analysis was performed using t-test (*p*<0.05) with SPSS V24.0 Software (IBM, Armonk, USA).

## Results

The t-test indicated a significant influence of the cement used (*p*< 0.000) and indicated a significant influence of surface pre-treatment in Fuji groups (*p*<0.002). While for the Vario groups (*p* >0.872) no significant influence was detectable in surface pre-treatment.

The highest measured values were found for Vario+AL (7,447 ± 0,945 MPa). The lowest scores showed Fuji +PM (3,803 ± 0,518 MPa) ( Table 2).

**Table 2:**
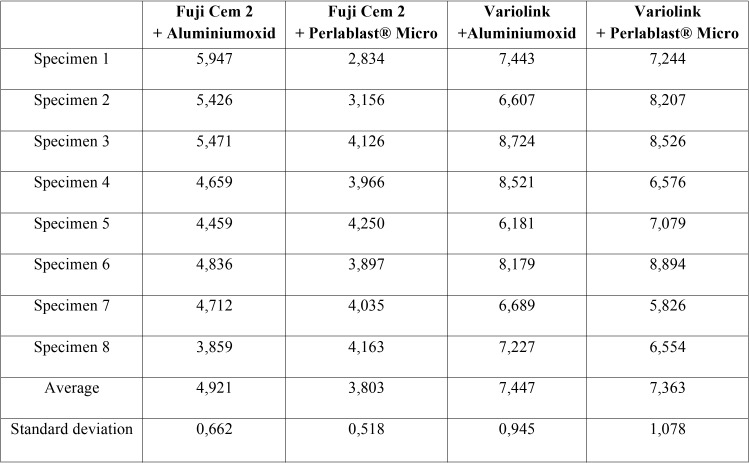
Shear bond testing [in MPa] of thirty-two 3D printed specimens.

Influence pre-treatment: The Fuji+AL group (4,921 ± 0,662 MPa) revealed significant higher shear bond strength than Fuji+PM (3,803 ± 0,518 MPa, *p*<0.002). No significant difference between Vario+AL (7,447 ± 0,945 MPa) and Vario+PM (7,363 ± 1,078 MPa) was found (*p*>0.872).

**Figure 2:**
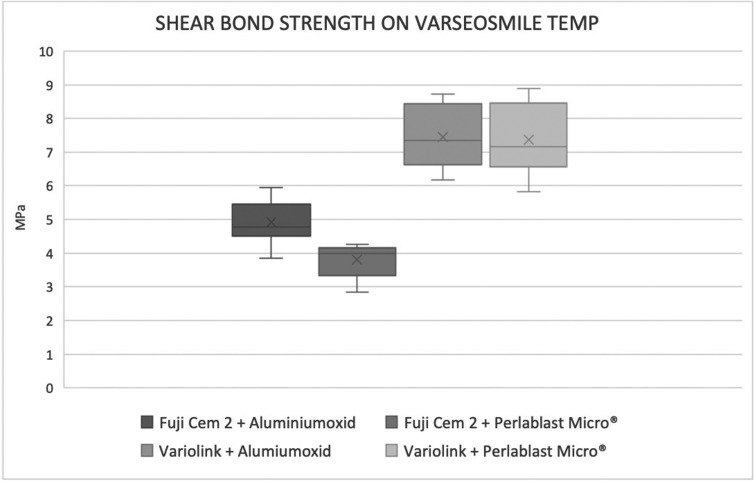
The four groups comparison.

Influence cement: The average shear bond strength among all Vario groups was significantly higher than Fuji groups (*p*<0.000). All test results are summarized in Fig. 2.

The surface analysis of the fractured specimens showed mainly substrate failures between the 3D printed bonded specimens for Fuji+PM, whereas mixed fractures and adhesive failures were predominately determined for Fuji+AL ( Table 3). Fuji groups revealed more adhesive failures than the Vario groups, where only substrate fractures of the specimens were detected. Nearly half of the specimens exhibited substrate fractures ( Table 3).

**Table 3:**
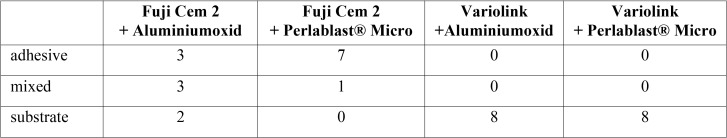
Splitting of the different ways of samples’ debonding.

## Discussion

The aim of this study was to analyse the influence of surface pre-treatment of 3D printed VarseoSmile Temp materials on the shear bond strength as well as the different shear bond strengths of resin cement and resin modified glass ionomer cement for cementation process. It was proved, Variolink® has a significant higher bond strength to VarseoSmile Temp than Fuji Cem 2. There was no significant difference between the two surface pre-treatments for Variolink® Esthetic, but the alumina group showed a little higher bonding strength. The resin modified glass ionomer cement Fuji Cem 2 showed a significant higher shear bond strength for alumina pre-treatment.

Regarding materials and methods, all specimens were 3D designed (Auto desk, Netfabb, San Rafael, CA, USA) and 3D printed (Varseo S, Bego, Bremen, Germany). The printed objects in the DLP process is dependent on the build angle, so all cylindrical test specimens were positioned at 180 ° to have the least possible variance between the test specimens and no supplies of printing an bonding surface (12). To compensate for variances of the cylindrical test specimens, the diameter was measured at 3 different points and the individual adhesive surface was calculated for each test specimen after the complete post-processing.

The study protocol followed the manufacturer’s instructions for the post processing. This ensured uniform adhesive surfaces. Surface pre-treatments were made manually in an angle of 45° and a distance of 1cm till the whole surface was blasted (6). Possible deviations in the distance or angle during the blasting process may possibly result in differences in the surface finish. Since later crown and bridge surfaces cannot be belabored at the perfect angle and distance, standardized radiation was not required. The position of the print-supports was placed on surfaces, which were not involved in the later cementing process. Thus, uniform adhesive surfaces were guaranteed without reworking with abrasives. Using mechanical testing machine Z010 (Zwick/Roell, Ulm, Germany) for reproducible forces (N=20) loaded on the specimens in the cementation process (8). An immediate cleansing with microbrushes during the bonding process after loading should prevent adulteration of the adhesive surface. A load of 6 minutes was chosen during the bonding process to ensure a secure setting of the materials.

Clinical use cannot be simulated entirely *in vitro* with standardized test, but it’s possible to find material-specific properties *in vitro* (13). The primary testing *in vitro* is important for fundamental understanding. Various methods are available to analyse the bonding strength of the cements. The most commonly used technique is the shear bond strength. By using the shear bond strength test, the mechanical testing of the specimens and cements used can be simply evaluated (14).

In the present study, significant difference between the pre-treatment methods for Fuji was found. This study is in line with Blixt *et al.* who found higher bond strength of surface pre-treatment with alumina. This effect is due to surface treatment with alumina results in increased roughness and surface enlargement, which provides greater physical and mechanical anchoring. Furthermore, the data have revealed that the shear bond strength of the Vario groups were statistically different compared to the Fuji groups. A possible reason for that could be a chemical interaction between components of Variolink® Esthetic and components of the VarseoSmile Temp. Further investigations should be performed to investigate this effect in depth.

As a limitation the bonding strength of the bonding area was technically not measured, because 18 out of 32 test specimens broke during loading, this means that it was not possible to determine the holding force precisely. The adhesion values for these bonding materials are expected to be even higher than the data obtained. For the statistical evaluation, the values at which a material failure occurred were evaluated in the same way as the values for adhesion loss. A different testing setup might clarify this effect for clinical relevance.

In the literature adhesion values for the cements can be found, with resin cements usually having higher values than resin modified glass ionomer cements. A study conducted by Piwowarczyk *et al.* showed higher shear bond strengths for resin cements than resin modified glass ionomer cements did on zirconia ceramic material (15). On alumina ceramics higher values were found for resin cements than for glass ionomer cements (16).

It is difficult to compare the shear strength values of the Fuji and Vario obtained in the present study to those of other similar protocols. This is due to the fact that every study is performed with different devices as well as with different operators. For this reason, the absolute data for bonding strength and the obtained values can be compared only inside the same study. In general, this study results are near to the study conducted by Peutzfeldt *et al.* (17). Several aspects, however, need further research. The resistance of this new prosthetic material for bridge restorations should be investigated to allow safe clinical use. It is not possible to predict safe fixation *in vivo* as there is currently no value which would need to be achieved *in vitro* in order to achieve this secure fixation. The assumed value of 20 MPa for secure fixation could not be refuted or proven to this day (13). Some more detailed studies are needed, especially chemical polymerization process within the printable resin need further investigation.

Another factor which might influence is the composite cements shrink during polymerisation, which may cause stress within the composite layer. They also undergo hydrolytic degradation and their coefficient of thermal expansion is different compared to natural tooth substance and ceramic materials (18-20). Furthermore, the effects of these parameters and possible interactions with VarseoSmile Temp should be analysed in future studies. As have a systematic *in vitro* investigation was performed as intra group comparison is reasonable.

## Conclusions

The findings showed a higher bond strength of Variolink® Esthetic compared to Fuji Cem 2 and a clear increase of bond strength for Fuji Cem 2 with alumina pre-treatment. Variolink® did not showed a significant difference between the two surface pre-treatments.

## References

[1] Dietrich H (2011). Temporäre Restaurationen als Schlüsselelement zur Erarbeitung der Ästhetik. Quintessenz.

[2] Burke FT (2005). Trends in indirect dentistry. Dent Updat.

[3] Stawarczyk B, Liebermann A, Kieschnick A (2017). Adhäsiv oder doch traditionell?. European Association of dental technology.

[4] Piwowarczyk A, Lauer HC, Sorensen JA (2004). In vitro shear bond strength of cementing agents to fixed prosthodontic restorative materials. J Prosthet Dent.

[5] Blixt M, Adamczak E, Lindén L A, Odén A, Arvidson K (2000). Bonding to Densely Sintered Alumina Surfaces: Effect of Sandblasting and Silica Coating on Shear Bond Strength of Luting Cements. Int J Prosthodont.

[6] Kohen D (2015). Einfluss Der Oberflächenvorbehandlung von PMMA-Basierten CAD/CAM-Kunststoffkronen Auf Die Verbundfestigkeiten Im Kronenabzugsversuch. https://edoc.ub.uni-muenchen.de/18156/1/Kohen_Daliah.pdf.

[7] Silva FL, Pamato S, Kuga MC, Só MVR, Pereira JR (2017). Bond strength of adhesive resin cement with different adhesive systems. J Clin Exp Dent.

[8] Mayerhöfer D (2012). Scherhaftkraft von drei selbstadhäsiven Kompositzementen auf bovinem Dentin und Zirkoniumoxid.

[9] (2012). GC FujiCEMTM 2. Inside Dentistry.

[10] Murmann EK (2010). Abrissfestigkeit von Kieferorthopädischen Bändern, Die Unterschiedlich Vorbehandelt Wurden. Ulm.

[11] Langrieger S (2010). In-vitro-Verbundfestigkeit von Zirkoniumdioxidkeramik und verschiedenen Befestigungskompositen nach unterschiedlicher Oberflächenkonditionierung Inaugural.

[12] Osman R, Alharbi N, Wismeijer D (2017). Build Angle: Does It Influence the Accuracy of 3D-Printed Dental Restorations Using Digital Light-Processing Technology?. Int J Prosthodont.

[13] Bayne SC (2012). Correlation of clinical performance with "in vitro tests" of restorative dental materials that use polymer-based matrices. Dent Mater.

[14] Sirisha K, Rambabu T, Shankar YR, Ravikumar P (2014). Validity of bond strength tests: A critical review: Part I. J Conserv Dent.

[15] Piwowarczyk A, Lauer HC, Sorensen JA (2005). The Shear Bond Strength Between Luting Cements and Zirconia Ceramics after two pre-treatments. Oper Dent.

[16] Begazo CC, De Boer HD, Kleverlaan CJ, Van Waas MAJ, Feilzer AJ (2004). Shear bond strength of different types of luting cements to an aluminum oxide-reinforced glass ceramic core material. Dent Mater.

[17] Peutzfeldt A, Sahafi A, Flury S (2011). Der Haftverbund von Zementen mit Dentin in Kombination mit verschiedenen indirekten Restaurationsmaterialien. Schweizerische Monatsschrift für Zahnmedizin.

[18] Silva EM da, Noronha-Filho JD, Amaral CM, Poskus LT, Guimarães JGA (2013). Long-term degradation of resin-based cements in substances present in the oral environment: influence of activation mode. J Appl Oral Sci.

[19] Ishikiriama SK, Maenosono RM, Oda DF, Ordóñez-Aguilera JF, Wang L, Mondelli RF (2013). Influence of Volume and Activation Mode on Polymerization Shrinkage Forces of Resin Cements. Braz Dent J.

[20] Bullard RH, Leinfelder KF, Russell CM (1988). Effect of coefficient of thermal expansion on microleakage. J Am Dent Assoc.

